# Measuring Vitality and Depletion During Adolescence: Validation of the Subjective Vitality/Subjective Depletion Scale in a Sample of Italian Students

**DOI:** 10.3390/pediatric17050098

**Published:** 2025-09-25

**Authors:** Giulia Raimondi, Michele Zacchilli, Christina M. Frederick, Fabio Alivernini, Sara Manganelli, Elisa Cavicchiolo, Fabio Lucidi, Tommaso Palombi, Andrea Chirico, James Dawe

**Affiliations:** 1 Department of Developmental and Social Psychology, Sapienza University of Rome, Via dei Marsi 78, 00185 Rome, Italy; giulia.raimondi@uniroma1.it (G.R.); fabio.alivernini@uniroma1.it (F.A.); sara.manganelli@uniroma1.it (S.M.); fabio.lucidi@uniroma1.it (F.L.); andrea.chirico@uniroma1.it (A.C.); 2 Department of Human Factors, Safety and Social Sciences, Embry-Riddle Aeronautical University, Daytona Beach, FL 32144, USA; frederic@erau.edu; 3 Department of Systems Medicine, Tor Vergata University of Rome, Via Montpellier 1, 00133 Rome, Italy; elisa.cavicchiolo@uniroma2.it; 4 Department of Dynamic and Clinical Psychology and Health Studies, Sapienza University of Rome, Via dei Marsi 78, 00185 Rome, Italy; tommaso.palombi@uniroma1.it; 5 Department of Humanities and Social Sciences, Universitas Mercatorum Telematic University, Pizza Mattei 10, 00186 Rome, Italy; james.dawe@unimercatorum.it

**Keywords:** vitality, depletion, self-determination theory, validation, measurement invariance, latent mean analysis

## Abstract

**Background/Objectives**: Adolescence is a critical developmental phase marked by rapid cognitive, emotional, and social changes that influence how individuals experience psychological energy and exhaustion. Self-Determination Theory recently proposed a dual-process model, based on two distinct, yet related, constructs: Subjective Vitality, associated with well-being and positive health outcomes, and Subjective Depletion, associated with illbeing and negative emotions. Since, to date, no study has investigated vitality and depletion during adolescence, this study aims to validate the Subjective Vitality/Depletion Scale (SVDS) in a large sample of adolescents. **Methods**: A total of 1111 Italian adolescents (Mage = 14.49, SDage = 1.49; 48% females) completed the SVDS and other validated self-report measures. Specifically, the psychometric properties of the SVDS across biological sex and age groups and latent mean differences across these groups were assessed. **Results**: Findings supported the dimensionality of the SVDS with two correlated factors, and its construct validity through associations with positive and negative affect and basic psychological needs satisfaction. Full invariance for the SVDS was achieved across biological sex and age groups. Latent mean analyses indicated that males reported higher levels of vitality compared to females (Cohen’s d = 0.46), with no significant differences for depletion; older adolescents reported lower levels of vitality (d = −0.23) and higher levels of depletion (d = 0.20) compared to younger adolescents. **Conclusions**: These findings support the SVDS as a valid and reliable instrument for assessing energy-related experiences in adolescence. The results suggest meaningful sex differences and a potential developmental trend of declining subjective energy from early to later adolescence.

## 1. Introduction

Adolescence is a critical developmental period marked by profound biological, cognitive, and emotional changes [1], often leading to heightened emotional reactivity and limited regulatory capacities [2]. In addition, this developmental phase is also associated with increased vulnerability to psychological distress and engagement in risk-taking behaviors, which can have lasting implications for health and well-being [3]. For these reasons, adolescence is considered a sensitive part of the individual’s life for both positive growth and potential negative development, making it a crucial phase for psychological assessment and intervention.

During the past decades, subjective vitality has stood out as a meaningful indicator of well-being [4,5]. Recently, Frederick and Ryan proposed, within the Self-Determination Theory (SDT), a dual-process model of energy regulation, which contributed to a deeper understanding of psychological functioning and well-being [6]. This model distinguishes between two distinct yet related constructs: Subjective Vitality (SV), which refers to the experience of feeling full of energy, alive, and having enthusiasm, and Subjective Depletion (SD), which refers to the experience of feeling drained, lifeless, and emotionally exhausted [6]. Several studies supported the positive protective role of SV, both in adolescents and adult population; for example, SV was found to be associated with greater engagement in self-directed, leisure-time physical activity [7], higher levels of motivation to learn [8,9], better sleep quality [10], healthy diet habits [11], positive relationships with both peers and teachers [12,13], satisfaction of basic psychological needs of autonomy, competence and relatedness [12,14,15], self-esteem [12,16], and self-efficacy [17]. Conversely, a decrease in SV has been related to low levels of motivation [8], greater frustration of basic psychological needs [14,18,19], depression [14,20], anxiety [14], and negative self-esteem [12].

On the other hand, the construct of SD has only recently been introduced by the Self-Determination Theory framework [12], and it has not yet been studied during adolescence. Building on Baumeister et al.’s [21] concept of Ego Depletion (i.e., temporary reduction in individual resources leading to a reduced capacity for self-control that occurs after prior acts of self-control), Frederick and Ryan [6] posit that SD occurs when an action is performed under internal or external pressures, and it is associated with ill-being and negative outcomes [6]. However, while the Ego Depletion model posits that any action that needs self-control (e.g., making a choice) will lead to energy depletion, Frederick and Ryan [6] reported a study by Moller et al. [22] that showed that when individuals felt pressured in their choices, there was a depletion effect on vitality and performance. However, when participants were given a true choice condition, the depletion phenomenon did not occur. Thus, whereas the ego depletion model assumes a fixed resource limitation, Self-Determination Theory offers a more dynamic process in which autonomously regulated actions foster SV and controlled regulation prompts SD. To date, however, there is no evidence regarding how SD manifests among adolescents, as previous validations have focused only on SV [23] or how it relates to basic psychological needs, key constructs of Self-Determination Theory [23,24,25,26]. Inconsistencies in the associations between need frustration and SV (e.g., [26,27]) suggest that low vitality does not fully capture adolescents’ risk-relevant energy states. Therefore, investigating SD alongside SV could offer a more comprehensive understanding of adolescents’ energy-related experiences and their implications for development and well-being.

Another majorly important aspect that has been severely neglected by existing studies on SV is whether energy-related levels may vary for adolescents of different age groups. Specifically, there are many cognitive, social, and emotional differences between early adolescence (i.e., till the age of 13 years old [2]) and adolescence (i.e., from 14 years old and on) that can alter the way SV and SD are experienced. For example, from a cognitive perspective, as individuals grow into adolescence, they develop advanced thinking abilities (e.g., abstract reasoning, problem-solving, perspective-taking), enabling more complex thought processes and the ability to debate ideas or opinions, compared to early adolescents [1,2,28]. Socially, they seek greater independence from parents and place increased importance on peer relationships, which become central to their emotional well-being [2,29]. Finally, adolescents tend to face increased academic pressures and social comparisons, challenging their mental health [30,31]. A further critical factor influencing early adolescents and adolescents’ well-being regards the transition from middle to high school [32,33]. This shift not only introduces students to new academic demands, but also significantly alters their social and emotional well-being and increases expectations for autonomy and motivation [32]. Taken together, all these aspects highlight adolescence as a particularly critical period for understanding whether SV and SD might change between early adolescents and adolescents.

Previous research has highlighted that SV is important for adolescents’ psychological development [34], helps them to cope with stress [35], and mitigates the impact of difficult life situations [36,37], such as the ones they have to face during adolescence. In contrast, depletion of energy removes a key self-regulatory resource and may increase the risk of negative outcomes [6]. Thus, validating an instrument that captures both SV and SD across early and later adolescence can help identify at-risk youth and assess whether SV and SD differ between these groups. However, because of all the differences between early adolescence and adolescence, it is essential to establish that the Subjective Vitality/Depletion Scale measures both SV and SD equivalently across these age groups before drawing substantive conclusions.

In addition to developmental differences, it is equally important to consider potential differences related to biological sex in how adolescents experience SV and SD. For instance, Bang et al. [38] found that boys reported significantly higher levels of vitality compared to girls in a large sample of elementary school children. More recently, Singh et al. [39] also reported biological sex differences in energy-related experiences among adults, showing that females tend to report lower vitality compared to males. However, the analyses conducted by both these studies [38,39] were limited to assessing *observed* means between groups, without testing for *latent* mean differences. This represents a crucial limitation, as it is a common practice in the field of social sciences to compare different group means using scale scores derived by summing or averaging item responses and, therefore, assuming that the observed means actually reflect the underlying latent construct for all groups considered [40]. Nevertheless, this assumption may lead to biased conclusions if the measurement is not equivalent across groups with different characteristics, such as the ones described in this study. For example, the same item might be interpreted differently between different age groups (i.e., early adolescents and adolescents), and males and females. Therefore, in order to make rigorous comparisons across groups, measurement invariance should be always assessed first. Measurement invariance is a statistical procedure that ensures that respondents from different groups interpret both the individual items and the underlying constructs in the same way [41]. This guarantees that differences in mean scores between groups reflect true differences in the constructs being measured, rather than biases or artifacts related to the instrument (e.g., variations in how questions are understood across groups). Therefore, measurement invariance should be established first before conducting valid and meaningful comparisons of latent constructs (e.g., SV and SD) across groups (e.g., age groups and biological sex).

To date, although some studies have examined SV in adolescents, no research has simultaneously considered both SV and SD within this age group. Furthermore, there is notable lack of information about the differences in SV and SD between early adolescents and adolescents, as well as between male and female adolescents. A key reason for this gap is the absence of studies validating the instruments that measure SV and SD in adolescents, as well as studies testing their measurement invariance across different those groups, which represents an essential step for making valid group comparisons. Therefore, the primary objective of this study is to validate the Subjective Vitality/Depletion Scale (SVDS), a measure capturing both positive and negative energy states, in a large sample of adolescence. Specifically, the first aim of this study is to examine the psychometric properties of the Subjective Vitality/Depletion Scale (i.e., factor structure, internal consistency and construct validity), assessing its measurement invariance across biological sex and early adolescents and adolescents. The second aim is to assess levels of Subjective Vitality and Depletion across these groups through a latent mean differences analysis.

In relation to the two aims of the study, we hypothesized the following:

**Hypothesis 1.**  *The factor structure will comprise two independent, yet negatively related, factors.*

**Hypothesis 2.**  *SV will positively correlate with the satisfaction of basic psychological needs and negatively with negative affect, whilst SD will correlate negatively with the satisfaction of basic psychological needs and positively with negative affect.*

**Hypothesis 3.**  *Given the differences in cognitive, social, and emotional development during adolescence, older adolescents will report lower SV and higher SD than early adolescents.*

**Hypothesis 4.**  *Considering the differences in energy level between males and females, females will show lower SV and higher SD than males.*

## 2. Materials and Methods

### 2.1. Participants

The current study analyzed data from a sample of 1131 students recruited from 12 lower and upper secondary schools located in northern, central, and southern Italy. This was a convenience sample, consisting of schools that agreed to participate in a larger research project on students’ well-being and school adjustment. After removing outliers, the final sample comprised 1111 students.

The mean age of participants was 14.49 years (SD = 1.49; age range: 12–17). Of these, 48% (537) were females, with 45% participants (501) classified as early adolescents and 55% participants (610) as adolescents. Overall, 53% students (585) attended lower secondary school (i.e., middle school), while 47% (526) attended upper secondary school (i.e., high school). The sample included 7% first-generation immigrants (79) and 6% second-generation immigrants (67). The distribution of parental education, a widely used indicator of socioeconomic status [42], was approximately normal (Skewness = 0.14; Kurtosis = −0.18).

For the early adolescence group, the age ranged from 12 to 13 years old, with a mean age of 12.94 (SD = 0.24), and 51% were females. The adolescent group ranged from 14 to 17 years old, with a mean age of 15.56 (SD = 0.94), and 46% were females.

For biological sex, among females, 48% were categorized as early adolescents and 52% as adolescents, and among males, 43% were categorized as early adolescents and 57% as adolescents. Among females, 54% attended lower secondary school and 46% attended upper secondary school, and among males, 51% attended lower secondary school and 49% attended upper secondary school. Descriptive statistics for both groups are reported in a table in the Appendix A (see Table A3).

In each participating school, research procedures were reviewed and approved by the School Board. Each student was given a letter explaining the aims of the study, which they showed to their parents in order to obtain their informed written consent. Participation remained voluntary, as students who had parental consent could still decline to take part.

Data were collected through an anonymous online survey administered at school during regular class hours. Although teachers were present in the classroom, to ensure participants’ privacy and the standardization of procedures, the administration of the questionnaire was managed by trained researchers. Before completing the questionnaire, students were provided with a standardized introduction explaining the purpose of the study, instructions for completion, and their right to withdraw at any time. To minimize missing responses, the online questionnaire required answers to all items; however, students were free to stop completing the questionnaire whenever they wished. The overall participation rate was 78%.

### 2.2. Measures

*Vitality and Depletion*: The Subjective Vitality/Depletion Scale (SVDS) [6,43] consists of two subscales that measures Subjective Vitality (item example: “*I have a lot of positive energy and initiative*”) and Subjective Depletion (item example: “*I feel drained*”), within the dual-process model of energy proposed by Frederick and Ryan [12]. Each subscale includes three items, which are rated on a 7-point Likert scale, ranging from 1 (“*Not at all true*”) to 7 (“*Very true*”). The Italian adaptation of the Subjective Vitality/Depletion Scale followed the procedure described elsewhere [42] (see Table A1 in Appendix A for the Italian version used in this study and the original version) and followed the guidelines for test adaptation from the Internal Test Commission [44]. The items’ suitability for these age groups was ensured by assessing comprehension in a subgroup of students, using the “*think aloud*” technique [45] in order to assess their adequacy in both early adolescents and adolescents.

*Psychological Well-Being*: The Feeling at School Scale (FASS) [46] was used to measure students’ positive and negative affect within the school setting (i.e., “*If you think about how you felt at school over the past few months, how often did you experience the following feelings?*”). The FASS contains 8 items (4 items measuring positive affect, e.g., “*happy, cheerful, good, calm*”; and 4 items measuring Negative Affect, e.g., “*sad, upset, worried, angry*”), rated on a 5-point scale, ranging from 1 (“*Never*”) to 5 (“*Very often*”). In the current sample, the Cronbach’s α for Positive and Negative affect were 0.87 and 0.79, respectively.

*Satisfaction of Students’ Basic Psychological Needs*: The Basic Psychological Needs Scale (BPNS) [47] consists of 3 subscales measuring the satisfaction of the basic needs of autonomy, competence, and relatedness, according to Self-Determination Theory. Each subscale includes four items assessing Autonomy Satisfaction (i.e., “*I feel a sense of choice and freedom in the things I undertake*”), Competence Satisfaction (i.e., “*I feel confident that I can do things well*”), and Relatedness Satisfaction. (i.e., “*I feel that the people I care about also care about me*”). Items are rated on a 5-point scale, ranging from 1 (“*Never*”) to 5 (“*Very often*”). In the current sample, the Cronbach’s α for Autonomy, Competence, and Relatedness Satisfaction were 0.70, 0.80, and 0.79, respectively.

### 2.3. Statistical Analyses

In a preliminary phase, we checked for the presence of outliers, which were identified through the Mahalanobis distance [48]. Subsequently, the normality of distribution of responses to the Subjective Vitality/Depletion Scale was examined by calculating the Skewness and Kurtosis of each item, with values within the range of ±2 supporting the assumption of normality [49]. The very small proportion of missing data (ranging from 0.1% to 0.6%) was handled using the Full Information Maximum Likelihood (FIML) method in Mplus. Finally, we assessed multivariate normality, through the Mardia’s skewness and kurtosis test [50].

In line with the first research aim, several analyses were conducted to examine the psychometric properties of the Subjective Vitality/Depletion Scale and to test its measurement invariance across groups. Confirmatory Factor Analyses (CFAs), with Maximum Likelihood Robust estimation method (MLR) [51] in order to account for possible violation of normality, independence, and homoskedasticity, were conducted to test the two-factor model and an alternative one-factor model. The following indices were used to evaluate model fit: (1) the chi-square (χ^2^) test, where p-values greater than 0.05 suggest an adequate fit to the data; (2) the Root Mean Square Error of Approximation (RMSEA), where values between 0.05 and 0.08 indicate an adequate model fit, while values of 0.10 or higher suggest poor fit [52,53]; (3) the Comparative Fit Index (CFI), where values above 0.95 signify good model fit and values of 0.90 or higher indicate acceptable fit [54]; (4) the Tucker–Lewis Index (TLI), with values above 0.95 indicating good model fit and values of 0.90 or higher representing acceptable fit [55]; and (5) the Standardized Root Mean Square Residual (SRMR), with values below 0.08 reflecting good fit [56]. Differences in χ^2^ were tested using the Satorra–Bentler scaled difference test, consistent with the use of the MLR estimator [50].

The Average Variance Extracted (AVE) [57] was measured to assess convergent validity, with values greater than 0.50 considered acceptable. Internal consistency reliability was analyzed through Cronbach’s Alpha (α) [58] and Composite Reliability (CR) [59], with values of α greater than 0.7 and CR greater than 0.6 are considered acceptable

A Structural Equation Model (SEM) was conducted to assess the construct validity of the Subjective Vitality/Depletion Scale by observing its correlation with other associated variables, specifically positive and negative affect, and satisfaction of basic psychological needs. All variables were entered into the model as latent variables. Before conducting the SEM, multicollinearity between basic psychological needs was assessed through the Variance Inflation Factor (VIF), with values above 10 indicating presence of multicollinearity [60].

Measurement invariance of the Subjective Vitality/Depletion Scale was conducted across biological sex (i.e., males vs. females) and age groups (i.e., early adolescents and adolescents) through a series of multigroup CFAs. Three levels of measurement invariance were considered [41]. First, configural invariance was assessed to determine whether the same number of factors, defined by the same items, fit the data equally across groups. Next, metric invariance was tested to evaluate whether each item contributed to its corresponding latent factor in the same way across groups. Then, scalar invariance was examined to assess whether item intercepts remained invariant across groups. To assess the adequacy of nested models representing different levels of invariance, ΔRMSEA (<0.015), ΔCFI (<0.01), and ΔSRMR (<0.01) were used in case of mixed measurement invariance results [61].

Regarding the second aim of this study, comparisons of latent mean differences were conducted in order to assess potential differences between males and females and between early adolescents and adolescents. In the analyses, the variances of the groups were constrained to be equal across groups so that Cohen’s d could be calculated. Importantly, conducting latent means comparisons, rather than using observed mean scale scores, offers a more accurate assessment of group differences because it accounts for measurement error and ensures that the comparisons are made between groups that interpret the constructs in the same way.

In line with methodological guidelines for SEM analyses [62] which require at least 250 subjects, the current sample size of 1111 students accounted for an adequate statistical power. Finally, all scales considered (i.e., SVDS, FASS, BPNS) were treated as continuous, in line with common practice in the literature [63]. All analyses were conducted with MPlus 8 [64], with the COMPLEX option to account for the nested structure of the data by classes, the Statistical Package for Social Sciences SPSS 26.0 [65], and with the *metan* package [66] for R 4.3.1 [67].

## 3. Results

Using Mahalanobis distance [48], we identified 20 outliers (1.77% of the sample); therefore, all the following analyses were conducted after removing these subjects from the sample (from 1131 to 1111). Regarding multivariate normality, Mardia’s skewness and kurtosis coefficients were significant (*p* < 0.001), indicating non-multivariate normality. Table 1 displays the reported means, standard deviations, skewness, and kurtosis values for each item of Subjective Vitality/Depletion Scale. All items’ skewness and kurtosis values ranged between −2 and +2, suggesting that the distribution of the responses was approximately normal.

**Figure 1:**
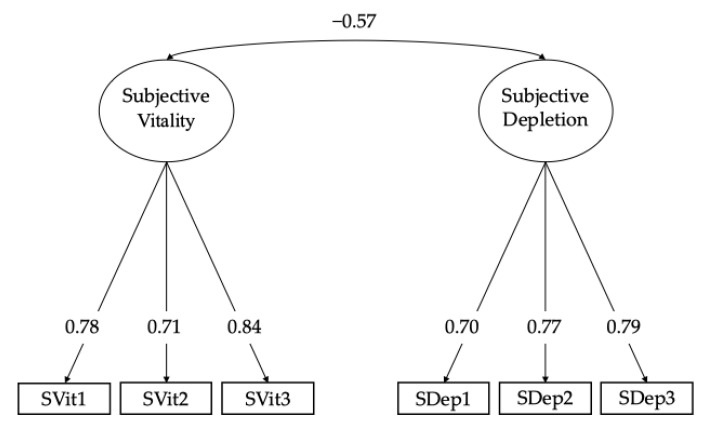
Factor structure of the SVDS. ***Note***: All factor loadings and correlation between factors are statistically significant, *p* < 0.001. Standardized factor loadings and correlation between factors are reported.

Regarding the first research aim, the CFA results indicated an optimal fit for the two-factor model of the Subjective Vitality/Depletion Scale (χ^2^ = 5.66 df = 8, *p* = 0.70; RMSEA = 0.00; CFI = 1.00; TLI = 1.00; SRMR = 0.009), with all factor loadings being statistically significant (*p* < 0.001). Standardized factor loadings and correlation between factors are shown in Figure 1 (see Table A2 in Appendix A for standardized factor loadings 95% Confidence Intervals, standard errors, and residual variances of all estimates of the scale). Meanwhile, the alternative one-factor model did not yield a good fit to the data (χ^2^ = 420.19 df = 9, *p* < 0.001; RMSEA = 0.20; CFI = 0.74; TLI = 0.56; SRMR = 0.10).

The Subjective Vitality/Depletion Scale reported optimal indices of internal consistency and convergent validity. Specifically, both dimension of Subjective Vitality and Subjective Depletion reported an AVE of 0.61 and 0.57, a Cronbach’s α of 0.82 and 0.79, and a composite reliability of 0.81 and 0.80, respectively. Discriminant validity was also supported according to the Fornell–Larcker criterion [46], as the AVE values for Subjective Vitality and Subjective Depletion were both higher than the squared correlation between the two factors (φ^2^ = 0.33).

The SEM with the correlations between vitality, depletion, and other constructs (i.e., positive and negative affect; satisfaction of the basic psychological needs autonomy, competence and relatedness) yielded an optimal fit to the data (χ^2^ = 620.92; df = 278; RMSEA = 0.03; CFI = 0.96; TLI = 0.95; SRMR = 0.03). Specifically, results indicated that Subjective Vitality showed positive correlations with positive affect (r = 0.60, *p* < 0.001, 95%CIs: 0.54–0.65), and the satisfaction of the three basic psychological needs (autonomy: r = 0.59, *p* < 0.001, 95%CIs: 0.53–0.66; competence: r = 0.65, *p* < 0.001, 95%CIs: 0.57–0.71; relatedness: r = 0.47, *p* < 0.001, 95%CIs: 0.41–0.53), and a negative correlation with negative affect (r = −0.38, *p* < 0.001, 95%CIs: −0.45–−0.29). In contrast, Subjective Depletion showed the opposite pattern. Specifically, Subjective Depletion was positively correlated only with negative affect (r = 0.58, *p* < 0.001, 95%CIs: 0.52–0.63), and negatively correlated with positive affect (r = −0.47, *p* < 0.001, 95%CIs: −0.54–−0.40) and the satisfaction of the three basic psychological needs (autonomy: r = −0.38, *p* < 0.001, 95%CIs: −0.47–−0.28; competence: r = −0.31, *p* < 0.001, 95%CIs: −0.40–−0.21; relatedness: r = −0.20, *p* < 0.001, 95%CIs: −0.28–−0.12). Finally, multicollinearity diagnostics indicated that none of the basic psychological needs exceeded the VIF value of 10 (autonomy: VIF = 2.843; competence: VIF = 2.657; relatedness: VIF = 1.375), indicating the lack of multicollinearity.

The measurement invariance analyses indicated that the model reached full scalar invariance (see Table 2) for biological sex (male vs. female) (χ^2^ = 30.99; RMSEA = 0.02; CFI = 0.99; SRMR = 0.03) and age groups (χ^2^ = 28.08; RMSEA = 0.01; CFI = 0.99; SRMR = 0.05).

Regarding the second aim, Table 3 shows the results of the latent mean analyses across the groups taken into account. Specifically, male students reported higher levels of vitality compared to female students, while no statistically significant mean differences were found for depletion; adolescent students reported lower levels of vitality and higher levels of depletion compared to early adolescent students (the observed mean scale scores, calculated as the average of the items within each scale, are presented in Table A2 in Appendix A). In terms of effect sizes, the difference in vitality between males and females was associated with a moderate effect size (Cohen’s d = 0.44), whilst the difference between adolescents and early adolescents in both SV and SD yielded small effect sizes (SV: Cohen’s d of = −0.25; SD: Cohen’s d of = 0.21).

## 4. Discussion

The first aim of the current study was to validate the Subjective Vitality/Depletion Scale (SVDS) in a large group of adolescents, assessing its psychometric properties and measurement invariance across biological sex and age groups.

In relation to the first hypothesis (HP1), the confirmatory factor analysis supported the hypothesized two-factor structure of the Subjective Vitality/Depletion Scale, reflecting Subjective Vitality and Depletion as distinct but correlated constructs. The model demonstrated an excellent fit to the data, with all items loading significantly onto their respective factors. This finding is consistent with theoretical assumptions within Self-Determination Theory, which conceptualizes vitality as the experience of feeling energized and alive, and depletion as a separate state of lack of energy and feeling exhausted, resulting from internal or external pressures [11,12]. Importantly, the internal consistency indices for both subscales were satisfactory, for both Cronbach’s α [58] and Composite Reliability [59] values, and Average Variance Extracted (AVE) [57] values supporting convergent validity. These results confirm that the Subjective Vitality/Depletion Scale is a reliable and valid tool to measure subjective vitality and depletion among adolescents, filling the gap of previous studies which focused only SV measures, primarily in adult populations [25,68,69]. The construct validity of the Scale was further supported by its correlations with psychological well-being indicators. In line with our second hypothesis (HP2), Subjective Vitality was positively correlated with positive affect and the satisfaction of basic psychological needs (i.e., autonomy, competence, and relatedness) and negatively correlated with negative affect. Conversely, Subjective Depletion showed the opposite pattern, being positively correlated with negative affect and negatively correlated with positive affect and need satisfaction. These results align with the Self-Determination Theory framework, which posits that satisfaction of basic psychological needs is associated with vitality and well-being, while their frustration contributes to depletion and ill-being [24,70]. Importantly, this is the first study to confirm these associations between SV and SD and basic psychological needs within adolescence, thereby extending previous findings, mainly conducted with adults, to a different developmental stage.

Moreover, measurement invariance analyses indicated that the Subjective Vitality/Depletion Scale was invariant across both biological sex and age groups, achieving scalar invariance. This finding is crucial, since it ensures that the respondents from different groups (i.e., females vs. males; early adolescents vs. adolescents) interpret both each individual item and the underlying constructs in the same way [41]. This result guarantees that the differences in mean scores truly reflect differences in the constructs being measured, rather than measurement bias or in how questions are understood across groups. Taken together, these results provide significant insights into the applicability of the dual-process model of energy proposed by Frederick and Ryan [6] in the school context.

In relation to our second aim, specifically the third and fourth hypotheses (HP3 and HP4), latent mean analyses indicated that male students reported higher levels of vitality compared to female students, while no significant differences emerged for depletion. This result is consistent with previous studies [38,39], which found higher vitality scores in males compared to females, in both children and adults. Bang et al. [38] suggested that biological sex differences might reflect different socialization patterns, which allow boys to express more energetic and active emotions, while girls tend to report higher negative affect. Moreover, these results further support the fact that SV and SD are indeed two related but distinct constructs, representing different aspects of the students’ energy experiences. However, the lack of biological sex differences in depletion in our study suggests that, despite males reporting higher vitality, both male and female adolescents experience similar levels of emotional and cognitive exhaustion. This finding indicates that while socialization may encourage males to express more energetic emotions, it does not necessarily protect them from feelings of depletion. Additionally, our study found that adolescents reported lower levels of vitality and higher levels of depletion compared to early adolescents (although the effect sizes were small; Cohen’s *d* of −0.23 and 0.20, respectively). This pattern may highlight the developmental challenges faced by adolescents. As individuals grow into adolescence, they may encounter increased academic pressures, identity-related concerns, and social comparisons, which can lead to reduced feelings of vitality and heightened experiences of depletion. These results align with the literature indicating that the transition from early adolescence to adolescence is often accompanied by greater psychological and academic demands that can negatively impact well-being [32,71].

### 4.1. Practical Implications

Taken together, all these findings have meaningful implications for interventions aimed at increasing vitality and decreasing depletion during adolescence.

For example, past studies suggested several interventions that can help enhance SV and decrease emotional exhaustion and fatigue, which are strictly related to SD. Indeed, interventions based on mindfulness and physical activity showed a positive effect on SV [72,73], and a negative effect on emotional exhaustion and fatigue [74,75]. These findings suggest that mindfulness programs not only mitigate emotional exhaustion and distress but also actively enhance SV, fostering a greater sense of energy and aliveness. By simultaneously reducing negative outcomes and strengthening positive psychological resources, such programs may contribute to improved academic performance and overall student well-being.

In addition, interventions should also aim at enhancing vitality, such as fostering autonomy-supportive environments, promoting positive meaningful relationships, and reducing external performance pressures, may be beneficial strategies to improve adolescents’ overall well-being. Practically, this can be achieved by providing meaningful choices, offering clear rationales, and promoting cooperative social interaction.

Conversely, practitioners should avoid pressuring language, conditional regard, and tasks assigned without explanation, which are likely to undermine vitality and increase depletion. However, some caution is needed. For example, supporting one’s own autonomy does not imply removing structure. On the contrary, it is still important to provide clear expectations, rationales, and bounded choices (autonomy-supportive structure). Moreover, choice can energize when it is made with few and meaningful options, but excessive or trivial options may increase self-control costs and invite depletion.

Finally, understanding depletion as a distinct construct can inform targeted interventions to alleviate adolescents’ feelings of exhaustion and emotional fatigue, which are often overlooked in well-being-based interventions.

### 4.2. Limitations

Despite the interesting results, some limitations of the current study should be acknowledged. Firstly, given the cross-sectional design of the current study, longitudinal studies are needed to further examine the stability of SV and SD over time, such as test–retest reliability, and their relationships with well-being and basic psychological needs in order to establish temporal ordering and causality.

Second, even though the current study included a large sample of adolescents, future studies are needed to generalize these results across different social and cultural contexts. Specifically, while there is strong evidence suggesting the applicability of Self-Determination Theory in different countries [76,77,78,79], research on the constructs of SV and SD is relatively new; therefore, the extent to which these findings can be generalized to other cultural contexts needs further exploration.

Thirdly, our study considered broad age categories (i.e., early adolescents and adolescents) and did not test for possible non-linear age trends. Therefore, future studies should examine the role of age with more fine-grained categories, as well as testing for non-linear age effects.

Fourthly, although SV and SD were measured using a scale that demonstrated good psychometric properties, the current study relied only on student self-reports, which may be subject to biases such as social desirability as well as common method variance. Future studies should address this limitation by conducting specific item analyses, such as Differential Item Analysis (DIF), and adopting multi-method approaches or developing new ones for measuring SV and SD instead of self-assessment methods. These methods could include qualitative interviews (e.g., brief semi-structured interviews to elicit recent episodes of feeling energized/depleted), behavioral methods (e.g., persistence/time-on-task, effort-based paradigms), or online behavioral tasks (e.g., persistence/effort–choice paradigm, a brief vigilance task, or a self-paced tapping task).

## 5. Conclusions

In conclusion, the present study provides robust evidence for the psychometric validity and reliability of the Subjective Vitality/Depletion Scale in a large sample of adolescents. The results support the two-factor structure of the scale, its measurement invariance across biological sex and age groups, and its construct validity through meaningful associations with positive and negative affect and the satisfaction of basic psychological needs. These findings suggest that vitality and depletion are distinct yet related constructs that can be effectively measured during adolescence. Given the importance of adolescence as a developmental stage, the Subjective Vitality/Depletion Scale represents a valuable tool for psychologists and researchers to assess adolescents’ subjective energy states in order to provide interventions aimed at fostering vitality and reducing depletion. Importantly, the latent mean differences across biological sex and age groups highlight the need for tailored strategies that consider the specific energy-related experiences of different subpopulations of adolescents.

## Figures and Tables

**Table 1:** Means, standard deviations, values of skewness, and kurtosis for each item of the SVDS.

Item Content	Mean	SD	Sk	Ku
1. I feel alive and vital (SVit)	3.32	1.13	−0.16	−0.67
2. I have a lot of positive energy and initiative (SVit)	3.29	1.13	−0.18	−0.67
3. I feel a sense of liveliness and spark (SVit)	3.47	1.12	−0.26	−0.71
4. I seem to have lost my “get up and go” (SDep)	2.32	1.19	0.66	−0.45
5. I feel drained (SDep)	1.98	1.19	1.14	0.40
6. I feel lifeless and unenthused (SDep)	2.04	1.12	0.99	0.29

***Note.*** SVit = subjective vitality; SDep = subjective depletion; SD = standard deviation; Sk = skewness; Ku = kurtosis. Items are rated on a 7-point scale, from 1 (“*Not at all true*”) to 7 (“*Very true*”).

**Table 2:** Goodness-of-fit indices for invariance of the SVDS across students with different characteristics.

Biological sex ^1^	χ^2^	df	RMSEA	CFI	SRMR	Δχ^2^	ΔRMSEA	ΔCFI	ΔSRMR
Configural model	9.899	16	0.000	1.000	0.011	-	-	-	-
Metric model	14.859	20	0.000	1.000	0.022	5.198	0	0	-0.01
Scalar model	30,901	26	0.018	0.997	0.036	17.679	−0.018	−0.003	−0.014
**Age groups ^2^**	**χ^2^**	**df**	**RMSEA**	**CFI**	**SRMR**	**Δχ^2^**	**ΔRMSEA**	**ΔCFI**	**ΔSRMR**
Configural model	10.002	16	0.000	1.000	0.013	-	-	-	-
Metric model	15.733	20	0.000	1.000	0.025	6.255	0	0	−0.012
Scalar model	27.914	26	0.012	0.999	0.048	14.015	−0.012	−0.001	−0.023

***Note.*** Χ^2^ = chi-square; df = degree of freedom; RMSEA = Root Mean Square Error of Approximation; CFI = Comparative Fit Index; TLI = Tucker–Lewis Index; SRMR = Standardized Root Mean Residual. χ^2^ differences were tested with the Satorra–Bentler scaled difference test. All χ^2^ are not statistically significant. ^1^ Females vs. males; ^2^ early adolescents vs. adolescents.

**Table 3:** Latent mean differences tests results.

	Subjective Vitality (95%CIs)	Subjective Depletion (95%CIs)	Cohen’s *d* Vitality	Cohen’s *d* Depletion
**Biological Sex**				
Female ^a^ vs. Male	0.393 *** (0.264–0.501)	−0.116 (−0.240–0.009)	0.46	-
**Age groups**				
Early adolescents ^b^ vs. Adolescents	−0.202 ** (−0.324–−0.080)	0.166 ** (0.043–0.289)	−0.23	0.20

***Note*.** 95%CIs = 95% confidence intervals. ^a^ Females are the reference group, with mean fixed to 0. ^b^ Early adolescents are the reference group, with mean fixed to 0. ** *p* < 0.01; *** *p* < 0.001.

## Data Availability

The data presented in this study are available on request from the corresponding author. (The data are not publicly available due to privacy restrictions.)

## Appendix A

**Table A1:** Original version [4] and the Italian version [43] of the SVDS.

		Original Version	Italian Version
Subjective Vitality	Item 1	I feel alive and vital	Mi sento pieno/a di vita
	Item 2	I have a lot of positive energy and initiative	Ho molta energia positiva e iniziativa
	Item 3	I feel a sense of liveliness and spark	Mi sento vivo/a e attivo/a
Subjective Depletion	Item 4	I seem to have lost my “get up and go”	Mi sento di aver perso la mia “voglia di fare”
	Item 5	I feel drained	Mi sento svuotato/a
	Item 6	I feel lifeless and unenthused	Mi sento spento/a e senza entusiasmo

**Table A2:** Standardized factor loadings with 95% confidence intervals, standard errors, and residual variances of all estimates of the SVDS.

		Factor Loadings 95% CI	Standard Error	Residual Variance (95% CIs)
**Subjective** **Vitality**	**Item 1**	0.74–0.82	0.021	0.39 (0.33–0.45)
	**Item 2**	0.67–0.75	0.024	0.49 (0.43–0.56)
	**Item 3**	0.81–0.87	0.018	0.29 (0.23–0.35)
**Subjective** **Depletion**	**Item 4**	0.66–0.74	0.024	0.51 (0.44–0.57)
	**Item 5**	0.72–0.81	0.026	0.42 (0.34–0.50)
	**Item 6**	0.74–0.83	0.027	0.38 (0.29–0.46)
		**Factor correlation 95% CI**	**Standard error**	
	**SV with SD**	−0.65–−0.49	0.041	

***Note*:** 95% CI = 95% confidence intervals; SV = Subjective Vitality; SD = Subjective Depletion.

**Table A3:** Scale scores of the SVDS across the groups.

	Subjective Vitality					Subjective Depletion				
	Mean (SD)	Median	IRQ	25°	75°	Mean (SD)	Median	IRQ	25°	75°
Males	3.58 (0.93)	3.67	1.33	3.00	4.33	2.02 (0.98)	2.00	1.67	1.00	2.67
Females	3.19 (0.93)	3.00	1.33	2.67	4.00	2.16 (0.94)	2.00	1.33	1.33	2.67
Early adolescents	3.50 (0.93)	3.33	1.33	3.00	4.33	1.99 (0.91)	1.67	1.33	1.33	2.67
Adolescents	3.36 (0.96)	3.33	1.33	2.67	4.00	2.16 (1.00)	2.00	1.33	1.33	2.67

***Note.*** IRQ = interquartile range; 25° = 25th percentile; 75° = 75th percentile. The observed mean scale scores are calculated as the average of the items within each scale. Standard deviation (SD) values are reported in parenthesis. Items are rated on a 7-point scale, from 1 (“*Not at all true*”) to 7 (“*Very true*”).
